# Microneedles: A New Generation Vaccine Delivery System

**DOI:** 10.3390/mi12040435

**Published:** 2021-04-14

**Authors:** Ipshita Menon, Priyal Bagwe, Keegan Braz Gomes, Lotika Bajaj, Rikhav Gala, Mohammad N. Uddin, Martin J. D’Souza, Susu M. Zughaier

**Affiliations:** 1 Vaccine Nanotechnology Laboratory, Center for Drug Delivery Research, College of Pharmacy, Mercer University, Atlanta, GA 30341, USA; Ipshita.Jayaprakash.Menon@live.mercer.edu (I.M.); Priyal.Bagwe@live.mercer.edu (P.B.); Keegan.Brazgomes@live.mercer.edu (K.B.G.); Lotika.Bajaj@live.mercer.edu (L.B.); uddin_mn@mercer.edu (M.N.U.); dsouza_mj@mercer.edu (M.J.D.); 2 Biotechnology Division, Center for Mid-Atlantic (CMA), Fraunhofer USA, Newark, DE 19711, USA; rikhav.praful.gala@gmail.com; 3 College of Medicine, QU Health, Qatar University, Doha P.O. Box 2731, Qatar; 4 Biomedical and Pharmaceutical Research Unit, QU Health, Qatar University, Doha P.O. Box 2731, Qatar

**Keywords:** microneedles, microneedle vaccine, vaccine delivery, skin vaccination, transdermal, immune response, microparticles

## Abstract

Transdermal vaccination route using biodegradable microneedles is a rapidly progressing field of research and applications. The fear of painful needles is one of the primary reasons most people avoid getting vaccinated. Therefore, developing an alternative pain-free method of vaccination using microneedles has been a significant research area. Microneedles comprise arrays of micron-sized needles that offer a pain-free method of delivering actives across the skin. Apart from being pain-free, microneedles provide various advantages over conventional vaccination routes such as intramuscular and subcutaneous. Microneedle vaccines induce a robust immune response as the needles ranging from 50 to 900 μm in length can efficiently deliver the vaccine to the epidermis and the dermis region, which contains many Langerhans and dendritic cells. The microneedle array looks like band-aid patches and offers the advantages of avoiding cold-chain storage and self-administration flexibility. The slow release of vaccine antigens is an important advantage of using microneedles. The vaccine antigens in the microneedles can be in solution or suspension form, encapsulated in nano or microparticles, and nucleic acid-based. The use of microneedles to deliver particle-based vaccines is gaining importance because of the combined advantages of particulate vaccine and pain-free immunization. The future of microneedle-based vaccines looks promising however, addressing some limitations such as dosing inadequacy, stability and sterility will lead to successful use of microneedles for vaccine delivery. This review illustrates the recent research in the field of microneedle-based vaccination.

## 1. Introduction

Infectious diseases have been prevalent in human history for centuries. The discovery and ongoing use of vaccines to prevent diseases have greatly benefited human health. As a result, many ailments have been eradicated, controlled, or deemed irreverent, allowing many generations of children to survive into adulthood, consequently increasing human life expectancy [1]. Vaccines mimic infections and utilize the immune system to produce immunity against the invading pathogen without succumbing to the pathogenesis of the disease. Traditionally, vaccines fall into three groups: whole pathogen vaccine, subunit vaccine, and nucleic acid vaccine [2]. First, whole pathogen vaccines have further subtypes; live attenuated vaccines are a type of whole pathogen vaccine that utilizes a weakened version of the pathogen to induce immunity while not being able to cause the disease. Inactivated vaccines are also whole pathogen vaccines that involve the inactivation of the pathogen using chemical or high-temperature treatments. Second, subunit vaccines focus on isolating and purifying specific components from the pathogen (or synthetic production) to induce immunity. Finally, nucleic acid vaccines involve introducing genetic material in the form of a plasmid DNA or messenger RNA that encodes for the antigens [2].

Vaccination is regarded as one of the most cost-effective medical interventions ever introduced. A publication from the Centers for Disease Control and Prevention estimates that between 1994–2013, vaccines prevented over 320 million illnesses, 21 million hospitalizations, and 732,000 premature deaths, saving at least $295 billion in medical costs [3]. However, the lack of licensed vaccines for emerging diseases, especially during epidemics and pandemics, can have a drastic impact on a country’s health and economy. The most recent example is the SARS-CoV-2 pandemic that has infected about 90.4 million people resulting in over 1.94 million deaths worldwide as of December 2020. The year 2020 saw the desperate need for an effective vaccine against SARS-CoV-2. Vaccines have always played an essential role in reducing the prevalence of infectious diseases worldwide. Therefore, continuous research and development on vaccine antigens and vaccine delivery are necessary for combatting future pandemics caused by novel and fast-evolving infectious diseases.

## 2. Vaccine Formulations and Their Delivery Methods

**Figure 1:**
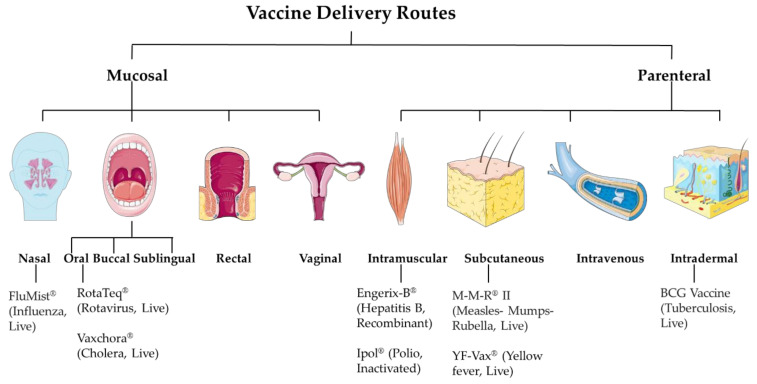
Vaccine delivery routes.

The targeted product profile of the vaccine is very important during its developmental phase. Factors such as vaccine antigen classes like live attenuated, inactivated, subunit, or most recently mRNA-based affect the selection of the vaccine dosage forms. The resulting formulation development ultimately affects its efficacy. While developing the formulation, a vital consideration factor is its intended route of administration. The essential balance between mucosal and systemic immune responses has always been an important consideration when deciding the vaccine delivery route. The mucosal immune response depends on tolerance, whereas for systemic immunity, it is the readiness of the immune system. The current approaches involve mucosal and parenteral delivery of vaccines (Figure 1). The mucosal sites include nasal, oral, buccal, sublingual, rectal and vaginal. And, the parenteral sites include intramuscular, subcutaneous, intravenous, and intradermal. The site of infection, transmission route, type of vaccine, and type of immune response expected are several factors that contribute to deciding the vaccine delivery route.

## 3. Mucosal Route

Mucosal vaccination involves vaccine delivery through mucosal sites such as nasal, oral, buccal, sublingual, rectal and vaginal. A large surface of the body is covered by mucosal tissues and is exposed to various infectious agents. Mucosal infections include respiratory tract diseases such as COVID-19, influenza, respiratory syncytial virus; sexually transmitted diseases such as gonorrhea and other genital tract infections; digestive tract infections such as rotavirus. Since many infections occur at mucosal sites, it is very important to develop strategies to neutralize these infectious agents at their site of entry. Thus, for localized immune response, mucosal immunization would be an attractive route, as it would mimic the natural infection [4]. Mucosal immunization also induces immune responses at other mucosal sites and/or systemically [5]. Furthermore, the mucosal interface contains well-organized lymphatic tissue, referred to as mucosa-associated lymphoid tissue (MALT). MALT includes both innate and adaptive arms of the immune system [6]. Mucosal vaccination has several advantages over the parenteral route, such as patient compatibility, does not require a healthcare professional, and does not generate any sharp waste [7]. Traditionally, mucosal immunization only focuses on the oral route. However, recently experimental animal models have been studied for sublingual, vaginal, and rectal routes. However, currently there are only oral and nasal licensed vaccines available in the market [8].

### 3.1. Nasal Route

Nasal vaccine delivery, a type of mucosal delivery, induces the nasal-associated lymphoid tissues (NALT) containing specialized M-cells to generate the immune responses, specifically innate immunity and IgA humoral and mucosal antibodies [9]. Nasal drops or sprays provide a non-invasive and pain-free alternative to conventional routes. The intranasal route requires lower doses of antigen and provides better antigen stability. Both mucosal and systemic immune responses are induced upon intranasal vaccination. Pulmonary vaccine delivery against measles and rubella have been studied [10]. Pulmonary vaccines include aerosol or dry powder inhaler systems. The dry powders can be reconstituted for nasal drops. Commercially, there is only one licensed nasal spray flu vaccine FluMist Quadrivalent^®^ (live attenuated influenza vaccine), that provides protection against influenza A (H1N1, H3N2) and influenza B [8].

### 3.2. Oral Route

Oral vaccination stimulates the immune system in the Peyer’s patch and mucosa-associated lymphoid tissue (MALT) in the gut wall [11]. It stimulates mucosal as well as systemic immune sites. The oral route is safe, patient compatible, easy to administer, and does not require a healthcare professional. However, oral vaccine development has challenges. Several antigens, which are protein in nature, would undergo degradation due to the harsh gut environment of mucosal enzymes. Thus, oral vaccine antigens lack stability. The oral route would require large antigen doses. However, the oral mucosal vaccine results in tolerance. The currently available oral vaccines include Cholera Vaccine Live Oral Vaxchora™ (PaxVax Bermuda Ltd.), Rotavirus Vaccine Live Oral Rotarix™ (GlaxoSmithKline Biologicals), Typhoid Vaccine Liv Oral Ty21a Vivotif™ (Berna Biotech, Ltd.), etc. These have shown to be effective in preventing the disease and priming the immune system. Prominent global oral vaccine manufacturers include GlaxoSmithKline Plc., Serum Institute of India Pvt. Ltd., Aventis (Sanofi S.A.), etc.

Particulate vaccines have several advantages over soluble antigens [12]. Yet, there is no licensed oral particulate vaccine. However, there is a firm understanding about the advantages of particulate vaccines at present [13].

### 3.3. Buccal and Sublingual Route

Recently, sublingual, and buccal routes (mucosal delivery in mouth) for vaccine administration are being explored. Sublingual mucosa includes the ventral area of the tongue and area under the tongue, buccal delivery incudes the cheeks, gums, upper and lower inner lips. These regions are rich in antigen-presenting cells like Langerhans cells, myeloid dendritic cells, and plasmacytoid cells [14]. Upon vaccination, the vaccine antigen will be captured by the APCs. APCs will then migrate to the draining lymph nodes. In the lymph nodes, APCs will interact with the CD4 and 8 T cells, thereby inducing the adaptive immune response. Vaccine delivery through these regions has advantages like those via the intranasal route. This route also requires comparatively lower amounts of antigen than with oral immunization [15]. Sublingual vaccination against influenza is found to protect against flu [16].

### 3.4. Rectal Route

To date, nasal and oral routes of mucosal vaccine delivery have been established. However, these vaccines, often subunits, require adjuvant combinations and there have been reported instances of neurological adverse reactions [17]. Therefore, to tackle these side effects, an alternative mucosal vaccine delivery route, the rectal route, has been proposed for the immunization against diverse microbial strains. Rectal vaccination against Chlamydia infection was found to provide protection following a challenge study [18].

### 3.5. Vaginal Route

Recent studies focused on the vaginal route of vaccine administration for genital infections and cancers such as the human papillomavirus (HPV) and cervical infection. In genital infections, topical application of a vaccine would be feasible for localized immune response. The genital mucosa generates specific immune responses after vaccination with inactivated and live-attenuated vaccines [19]. Another study demonstrates outer membrane vesicles (OMV) with T-helper cells driving adjuvants and interleukin-12 intravaginal vaccine approach against gonorrhea [20]. These studies are successful on experimental animal models that are not yet applied in human. However, there will be an anticipated problem of patient incompatibility with vaginal route for vaccine delivery.

## 4. Parenteral Route:

**Figure 2:**
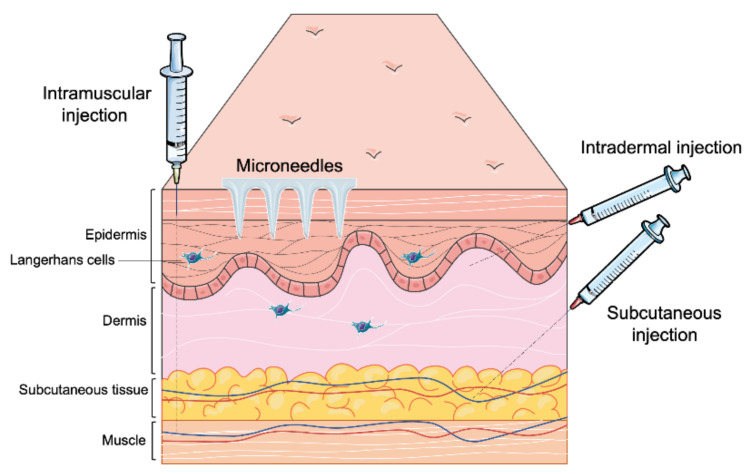
Different parenteral routes of vaccine administration.

Most of the vaccines available in the market are administered through the parenteral route (Table 1) [21]. Recently developed vaccines against COVID-19 are also delivered through the parenteral route. This route offers various approaches (Figure 2) such as intramuscular (IM) slow sustained release), subcutaneous (SC) (slow sustained release), and intradermal (targeting antigen-presenting cells in the dermal region) [22]. Depending upon the type of immune response desired, the vaccine can be delivered to the dermis, muscle, SC region, or veins [23]. Burst release of antigen can be helpful to induce an innate immune response. However, the current research focuses on needle-free delivery systems which provide longer time of slow sustained release of antigens and more priming to the innate immune system leading to optimal adaptive immune responses. A sustained release of antigens is desirable as it ensures that the APCs identify and take up the antigens over a long period of time to ensure a robust adaptive immune response. Additionally, microneedles reduce needle-stick injuries, requiring a medical professional and biohazardous waste [24].

### 4.1. Intramuscular Route

Intramuscular vaccines are administered into the muscles, the vastus lateralis (anterolateral thigh, recommended in infants), and the deltoid (upper arm). Inactivated vaccines are generally administered via the IM route. The recently developed Pfizer-BioNTech and Moderna COVID-19 vaccines are administered through the IM route [25,26,27]. These novel mRNA-based vaccines typically induce an excellent systemic immune response involving IgM, IgG and IgA antibodies. IM immunization does not induce very high mucosal immunity in the form of IgA. However, the efficiency of vaccine dose is higher in terms of IM injection.

### 4.2. Subcutaneous Route

Subcutaneous vaccines are administered under the skin. The vaccine is injected between the skin and the muscle. Live-attenuated vaccines (e.g., MMR, yellow fever) are administered via the SC routes.

### 4.3. Intravenous Route

Intravenous vaccine delivery is the fastest delivery. The vaccine is administered into the veins directly. This route is recently being explored for developing a vaccine against tuberculosis [23]. In the past, researchers have studied this route for developing an anti-malarial vaccine. However, this route has a major drawback that the vaccine antigen is rapidly cleared from circulation and it should be administered by a trained professional.

### 4.4. Intradermal Route

Intradermal route of vaccination is most used for immunization against Tuberculosis Bacille Calmette-Guerin (BCG) and rabies [28]. The intradermal region is rich in antigen-presenting cells, with dermal dendritic cells and Langerhans cells in the epidermis. Alternative devices for vaccine delivery are being developed. Needle-free disposable syringe injectors can reduce needle-stick injuries. Microneedles, vaccine-loaded small micron-sized needle patches, are being studied and can avoid cold chain storage [29]. Compared to the conventional syringe needle, microneedles’ smaller size allows for precise delivery into the dermal layers. The insertion depth is sufficiently shallow to avoid impinging innervated tissue, resulting in a painless injection and reduced needle phobia in patients.

## 5. Microneedles for Transdermal Delivery

**Figure 3:**
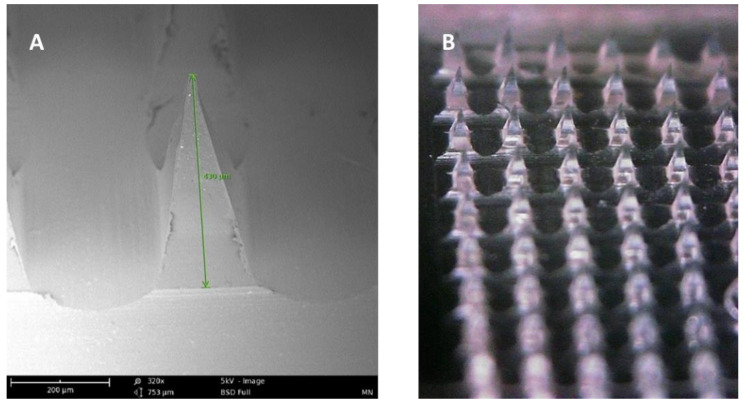
(**A**) SEM image of a dissolving polymeric microneedle (size: 430 μm, scale 200 μm). (**B**) Optical microscope image of the same microneedle array patch.

Microneedle arrays are minimally invasive micron-sized needles that penetrate the stratum corneum, which is the skin’s primary barrier to delivering a therapeutic through the skin. Microneedles vary between 50–900 microns in height (Figure 3) and are fabricated using various geometries and various metals, silicones, and polymers. The application of microneedle patches into the skin forms microscopic aqueous pores to allow the diffusion of drugs to the skin’s epidermal layer [30]. The concept of microneedles was first established decades ago but only became prominent in significant research in the mid-1990s. In contrast to hypodermic needles, microneedles are more patient compliant as they are pain-free and can be self-administered. Microneedles are micron-sized as to be able to deliver almost any drug or small particle formulation but not long enough to cause any pain during administration (Appendix A Table A1). Additionally, microneedle delivery allowed delivery to precise tissues such as within the skin or the eye. There are several types of microneedles: solid, coated, dissolving, and hollow [31].

### 5.1. Solid Microneedles

Solid microneedles are often used as a skin pretreatment. They are inserted into the skin and then removed to form micron-sized pores on the skin surface. Drug solutions within a patch can then be applied to the surface, which contains the micropores. Another variation utilized a roller containing solid microneedles, which pokes holes in the stratum corneum multiple times as the roller moves across the skin [32].

### 5.2. Hollow Microneedles

Hollow microneedles are miniature versions of the conventional hypodermic needles. Drug delivery through hollow microneedles is achieved through a pressure-driven flow of a liquid formulation. In contrast to other types, hollow microneedles are challenging to produce due to their structure and fragility [32]. However, hollow microneedles can deliver large, continuous amounts of actives compared to the other microneedle types [30].

### 5.3. Dissolving Microneedles

Dissolving microneedles are made using biodegradable materials such as various polymers and sugars loaded with therapeutics. After the needle is applied to the skin, the needles dissolve to release the payload into the skin. The advantage of dissolving microneedles in contrast to solid and hollow microneedles includes the ease of fabrication and single-step application of the patch. Dissolving microneedles have been looked at extensively for delivery vaccines through the skin [32].

### 5.4. Coated Microneedles

Coated microneedles consist of solid microneedles that have been coated with a drug solution or dispersion. There are various methods to produce coated microneedles, including dip coating, in which the microneedles are “dipped” into the coating solution. Spray coating can also be used to coat the needles. Coated microneedles are not as commonly used as solid, hollow, and dissolving microneedles since they offer a minimal amount of surface area for drug absorption.

## 6. Composition of Microneedles

Various materials can be used to produce microneedle arrays and these materials are all FDA approved [33]. The first material used was silicon. Advantages of using silicon include high flexibility to allow needles with customizable shapes and sizes. However, silicon microneedles’ main limitations include high cost, long fabrication times, and multi-step processing [30]. Metals can also be used for microneedle fabrication. Some common materials include stainless steel and titanium. Metal microneedles are considered biocompatible as metals have been used in medical devices for decades. Additionally, metals produce desirable mechanical properties. Ceramics have also been used to make microneedles. They can be produced at a lower cost compared to other materials. Silica glass can be used to make microneedle patches with varying needle geometries quickly in a small-scale setting.

However, silica glass can be brittle and generally has be to be produced by hand. Carbohydrates can be easily formulated into microneedle patches through molding of hot melts/slurries of the carbohydrates. These patches dissolve upon skin insertion and are cheap to manufacture and safe for use in humans. Maltose, trehalose, sucrose, and mannitol are some of the sugars that have commonly been used to formulate dissolving microneedles. The main disadvantage of these types of needles is the high-heat treatment needed to produce these needles, limiting therapeutics that can be incorporated. Additionally, the integrity of these needles can be drastically affected by temperature and humidity. More commonly, FDA approved polymers are used to fabricate microneedles. Moreover, polymers such as polyvinyl alcohol (PVA), polyvinyl pyrrolidone (PVP), hyaluronic acid, polylactic acid are some of the few polymers used extensively in the fabrication of dissolving microneedles [33,34]. Many polymers are biocompatible, biodegradable, possess low toxicity, are mechanically strong enough to penetrate the skin, and are low cost [30].

## 7. Vaccination Using Microneedles

**Figure 4:**
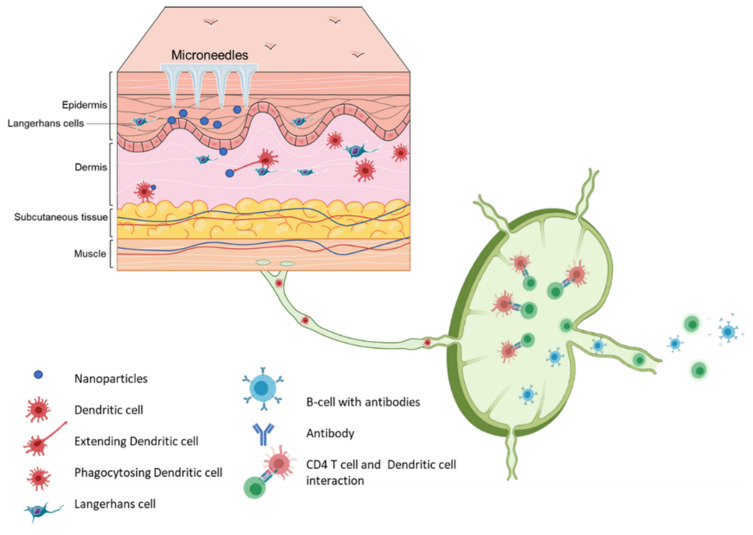
Schematic representation of immune cells activation post immunization using microneedle vaccines.

Vaccination via the skin or the intradermal route of administration was the original concept of immunization. Edward Jenner, who discovered the first vaccine (against smallpox), administered the vaccine by scratching it onto the first vaccine recipient’s skin [35]. The most common vaccination routes are the IM route or the SC route employing a painful needle for administration. Currently, only the Bacille Calmette-Guérin (BCG) and rabies vaccines are administered via the intradermal route [28]. The skin has an abundant presence of antigen-presenting cells (APCs) in the form of Langerhans cells and dermal dendritic cells (Figure 4). As a result, the skin as a site of vaccination is gaining importance. Microneedles as a form of transdermal administration were developed in 1976; however, this concept was used for immunization only in recent years [36]. Soluvia™ was the first intradermal vaccine developed by Becton, Dickinson, and Company. The microneedle system is comprised of a 30-gauge metallic microneedle that is inserted 1.5 mm into the skin, which allows the delivery of the vaccine antigen into the dermis. This system was used to deliver the influenza vaccine [37] (Appendix A Table A2). Since then, microneedles have been explored extensively for vaccination against various viral and bacterial infections and immunotherapy for cancers (Appendix A Table A3).

### 7.1. Solid Microneedles for Vaccine Delivery

As described earlier, there are different kinds of microneedles; solid, hollow, and dissolving. Each of these microneedle systems has been investigated as immunizations tools. Stainless steel is the most common material used to make solid microneedles for vaccine delivery [31,38]. These stainless-steel microneedles dip-coated with various antigens, including antigen solutions as well as antigens encapsulated in nanoparticles [39,40]. The coated antigen gets released into the skin layers upon administration of metal microneedles. As a result, the coating of the microneedles in one of the key aspects which determines the efficacy of metal microneedle vaccine. Thus, there have been advancements to improve the efficiency of stainless steel microneedles, such as nanopatterning the stainless-steel microneedle surface [41]. This nanopatterning has been shown to enhance the dip coating of antigens onto the microneedle tips by improving the hydrophilicity of the microneedle surface. Such a nano-patterned microneedle system showed enhanced plasmid DNA loading. This enhanced loading was responsible for the improved immune response observed in the in vivo study compared to conventional metal microneedles [41]. Different methods have been used to enhance the coating of antigens on the surface of solid microneedles. Solid microneedles coated with multilayers of charge reversal pH-sensitive copolymers enhanced the delivery of the DNA vaccine to antigen-presenting cells, thus producing enhanced immune response [42]. The Mantoux technique is the traditional intradermal administration method; however, an improved or first-generation intradermal administration technique involving solid microneedles in a Nanopatch™ comprising 10,000 micro projections/cm^2^ each 250 µm long enhanced the antigenicity of the HPV vaccine administered. Moreover, this method allowed administering the HPV vaccine without the adjuvant with a transfer efficiency of almost 20% [43]. The Nanopatch^TM^ was also used in a clinical trial to deliver an influenza vaccine coated on the microneedles. The randomized, partly-blinded, placebo-controlled study reported that most of the subjects preferred the microneedle vaccine over their past IM injection experience [44]. Moreover, other novel materials have also been explored to fabricate solid microneedles for administering vaccines for infectious diseases and cancer immunotherapy [45]. A solid microneedle system was developed using silk fibroin for immunization against influenza, *Clostridium difficile*, and *Shigella*. A pre-clinical study in mice demonstrated that silk fibroin was able to form solid microneedles, which provided long-term protection with dose sparing effect in case of influenza and provided moderate protection from challenge with *Clostridium difficile* [46]. The authors note that the amount of antigen delivered to the mice upon administration of the vaccine is less than the coated dose. The difference in dose highlights one of the challenges of vaccination using microneedles [46].

### 7.2. Hollow Microneedles for Vaccine Delivery

Efficient targeting of APCs is imperative to achieve an effective immune response. It is noted that particulate vaccines are more efficiently taken up by the APCs [47]. Combining the effective targeting of APCs using the particulate vaccine and pain-free administration of the vaccine offers a powerful tool for immunization. As a result, a lot of research is now focused on delivering antigens encapsulated in a polymeric particle using microneedles. Hollow microneedles are studied extensively to deliver particle-based vaccines. These microneedles contain the vaccine antigen, filled inside the hollow needles which upon administration, deliver the vaccine antigens in the skin. A microneedle system comprising of applicator-controlled silica hollow microneedles facilitated the delivery model antigen ovalbumin with and without adjuvant encapsulated in various optimized nanoparticles, namely poly (lactic-*co*-glycolic) (PLGA) nanoparticles, liposomes, mesoporous silica nanoparticles (MSNs), and gelatin nanoparticles (GNPs). Penetrating a depth of 120 microns, the microneedle delivery of PLGA nanoparticles and liposomes induced an excellent humoral and cellular immune response [48]. Similar results were observed when ovalbumin with and without adjuvant encapsulated in PLGA nanoparticles was administered using 3M plastic hollow microneedles attached to an applicator [49].

Hollow microneedles have also been used to deliver DNA vaccines encapsulated in a nanoparticle system. The DNA vaccine encoding for ovalbumin was encapsulated in cationic niosomes to produce a better and more robust immune response than the naked DNA. Moreover, the DNA vaccine encapsulated in the niosome resulted in a better immune response than the SC route [50]. Therapeutic cancer vaccine administered using a digitally controlled hollow microneedle injection system required significantly less antigen as compared to traditional intradermal injection. This unique hollow microneedle system composed of silica achieved automated micro-injections (0.25–10μL) to deliver a synthetic long peptide HPV E7 [43,44,45,46,47,48,49,50,51,52,53,54,55,56,57,58,59,60,61,62,63] derived from HPV encapsulated in cationic liposomes. There microinjected antigen induced a robust cell CD8+ cytotoxic and CD4+ T-helper cell response [51].

### 7.3. Dissolving Microneedles for Vaccine Delivery

Even though hollow microneedles are efficient in inducing a robust immune response, they still leave material on the skin and are not made of biodegradable material. Dissolving microneedles offer advantages over these shortcomings. Dissolving microneedles are composed of FDA approved polymers and can be loaded with the vaccine antigen or nanoparticles containing the vaccine antigen. Upon administration, these microneedles dissolve completely to release the vaccine into the skin. Dissolving microneedles loaded with microparticles have the advantage of the slow release of antigens to as to achieve sustained release of antigen which helps in achieving a robust adaptive immune response. Dissolving microneedles for immunization was first developed for the administration of the influenza vaccine. The microneedle system was composed of polyvinyl pyrrolidone and could deliver the encapsulated lyophilize antigen in 5 minutes [52]. Since then, several dissolving microneedles for vaccines have been fabricated with various polymers and sugars [53]. Dissolving microneedles have also been proven to maintain the antigen’s stability at room temperature (25 °C) for more than one year. Influenza vaccine in dissolving microneedles composed of polyvinyl alcohol (PVA) and sucrose maintained its stability at 25 °C and 60 °C for up to 24 months and four months, respectively [54]. Similarly, a malaria antigen had superior stability in a dissolving microneedle patch composed of sugars as compared to the vaccine in its liquid form [55]. Furthermore, Dissolving microneedles made of PVA have been widely studied and have proven to elucidate significantly robust immune responses upon challenge. They have also been an efficient delivery system for prophylactic DNA vaccine for cervical cancer [56,57]. Protective immune responses in pregnancy have also been studied upon challenge with tetanus toxin compared to traditional routes such as IM [58]. PVA microneedles have demonstrated induction of T_H_1 cytokines (IFN-γ and IL-12) when challenged with *Streptococcus suis* bacteria and confer long-term protection by induction IgG2a antibodies specific to *S. suis* bacteria [59]. Dissolving microneedles have proven to induce superior antibody response against adenovirus-based *Plasmodium falciparum* malaria vaccine, AdHu5–PfRH5. A study proved that low prime dose given using dissolving microneedles, and a boost dose given intramuscularly gave a robust immune response as compared prime dose given intramuscularly. Thus, microneedle aid in achieving a superior immune response as compared to the conventional intramuscular route [55]. Additionally, microneedles composed of PVA and sugars demonstrated also enhanced immune response upon challenge with *Neisseria gonorrhoeae* [29]. Like hollow microneedles, dissolving microneedles have also been used to study the delivery of vaccine antigens encapsulated in cationic liposomes. The cationic liposome-based microneedle vaccine protected against a challenge with the bacteria *Leishmania donovani* [60]. The BCG vaccine is an intradermal vaccine; however, it is administered using a hypodermic needle. A study involving coated BCG vaccine coated on dissolving microneedles made using sodium alginate and sugars revealed that the microneedle system produced an immune response comparable to the injected vaccine [61].

Hyaluronic acid is another biodegradable polymer that is used a lot for the formulation of dissolving microneedles for vaccine delivery for infectious diseases and cancer immunotherapy [62]. A canine influenza vaccine was successfully coated on hyaluronic acid microneedle tips. The microneedle had separable tips in a system called insertion-responsive microneedles. The microneedle coated with freeze-dried vaccine provided thermal stability to the vaccine when stored at 50 °C for three weeks compared to the liquid form and protected from challenge with H3N2 wild type virus [63]. Another study for insertion responsive microneedle system for canine influenza vaccine proved that the system could be used to vaccinate dogs without the need to shave their hair and provided better compliance for the dog and owners [64]. Hyaluronic acid microneedle induced a robust immune response with just one dose in a B16F10 mouse melanoma model compared to microneedles without degradation trigger or intratumoral injection of free, programmed death-1 with the same dose for skin cancer [65]. Sustained-release polymers have also been employed for the formulation of microneedles for vaccine delivery. A microneedle system comprising poly (lactic-*co*-glycolic acid) was used to prepare vaccine cores and shells using the micro-molding technique. This system was used to deliver the clinically approved vaccine Prevnar-13 against the bacterium *Streptococcus pneumoniae*. It was observed that the microneedle vaccine was able to induce an immune response comparable to that obtained on multiple SC injections [66].

Additionally, a hepatitis B vaccine formulated in a dual release pattern using polylactic acid and carboxymethylcellulose (CMC) could function as a prime and booster in one microneedles system. The microneedle vaccine was again able to produce an immune response similar or even higher than two shots given using the conventional administration method [67]. As a result, microneedles made using sustained-release polymers with various degradation kinetics can be adopted to achieve vaccines that require multiple boosters. Additionally, dissolving microneedles have been extensively explored as administration systems for antigens encapsulated in micro and nanoparticles [68]. Microneedles have also been used to deliver combination vaccines in the form of a compartmental microneedle array (CMA). This CMA formulated with polylactic acid consisted of two separate sections in the same microneedle patch. The two sections were coated with B-Y influenza vaccine for B/Yamagata virus B-V influenza vaccine for B/Victoria virus. The in vivo studies in mice indicated that the mice vaccinated with the CMA had higher neutralizing antibodies and better survival rates than the traditional IM route. The dissolving microneedle influenza vaccine has also been reported to have better patient compliance than the conventional IM route in Phase I clinical study [69]. Dissolving microneedles have also been studied as a potential vaccination system to tackle the COVID-19 pandemic. Researchers found that microneedles formulated using CMC coated with the SARS-CoV-2-S1 protein produced significant levels of antigen-specific antibodies [70].

## 8. The Barriers to Microneedles-Based Vaccines

**Figure 5:**
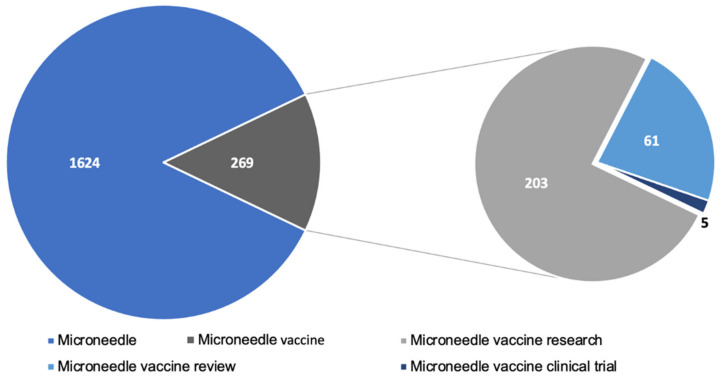
Microneedle related literature published in PubMed since 2017.

A significant amount of research has gone into studying microneedles for vaccination. A PubMed search with the keyword ”microneedle vaccine” yielded 236 articles published since the year 2017. The articles included pre-clinical and clinical research articles as well as review articles. Figure 5 depicts the breakdown of recent research published. Pre-clinical studies show very promising results indicating that microneedles are comparable or even better than conventional injectable vaccines. However, there are few clinical studies conducted in humans. A quick search in clinicaltrias.gov with keywords’ microneedle vaccine’ revealed that one Phase 1 trial with 100 participants and another trial with 33 participants is completed and has results. Apart from these, there are seven other trials for the microneedle-based vaccine at various stages of clinical trial. Moreover, the research trend in the field of microneedles is increasing rapidly and has been reviewed elsewhere [71]. This indicates that the clinical translation of microneedles is one of the challenges for microneedle-based vaccines. The lack of clinical data also implies that the scale up of microneedles is another aspect which is a barrier to clinical translation of microneedle vaccines. As a result, large scale production of microneedles will require a lot of in depth considerations to adopt it to industrial scale and are discussed in other reviews [72,73,74]. Vaccines are the most effective approach to fight infectious diseases, and the COVID-19 pandemic has increased the need for improving vaccination compliance. A lot of people avoid getting vaccinated due to the fear of the needle. Microneedle vaccination is pain-free and will have better patient compliance.

Microneedles do not need cold-chain storage; thereby, this is another area where microneedles may improve vaccine coverage in developing countries and places where cold chain storage may be a hurdle to vaccination. The thermostability of vaccines in a microneedle patch needs to be studied extensively to reach this goal of vaccines not requiring cold-chain storage. As per WHO guidelines, microneedles-based vaccine drug products need to have stability data for at least Vaccine vial monitor (VVM) category type 7 or beyond. A higher VVM category such as VVM 14 or VVM30 would be ideal for room temperature-based vaccines. The achievement of such stability would be possible with microneedles due to the dry and amorphous nature of the microneedle matrix [75]. Likewise, microneedles can be self-administered; however, it is imperative to design microneedles so that variation in administration pressure by different individuals won’t affect the dosage form’s efficiency.

The mass immunization program is the only solution to battle a pandemic such as COVID-19. Microneedle vaccines have shown a lot of promise in various pre-clinical studies for COVID-19 [70,76,77,78]. Moreover, several companies are developing microneedle-based vaccines for COVID-19 and various other infectious diseases and cancers (Table 2).

Vaccines for human application will need to be sterile and free of pyrogens except the vaccine antigens. Regulatory acceptance of microneedles-based vaccines will improve if sterility is addressed. Most vaccines are sterilized using filtration; however, as microneedles are solid patches that cannot sterilized using sterile filtration, different sterilization methods may need to be used. Furthermore, a microneedle patch containing a vaccine may lose its antigenicity if it is sterilized using radiation. As a result, microneedles may need to be manufactured in aseptic conditions to maintain sterility. This may pose an additional hurdle in the bulk manufacturing of microneedle vaccines to combat pandemics that require mass vaccination. From a regulatory point of view, microneedle vaccines will be considered a combination product under FDA 21 CFR 3.2. Combination products are defined as products composed of two or more regulated products that are physically, chemically, or otherwise combined to serve as a single entity [93]. The regulatory considerations for microneedles include needle length, sharpness, ability to penetrate living layers of skin, degree of control of administration (manual or mechanized) if they are considered medical devices [94]. Microneedle vaccines will require regulatory submissions for both the vaccine and the microneedle and the combination product [95].

## 9. Conclusions

Microneedles are a very powerful drug delivery system for immunization awed to their slow sustained release of vaccine antigens. They eliminate the need for a skilled person to administer the vaccine. They can essentially be self-administered, providing a potential for an attractive system for large-scale immunizations during pandemics. Apart from being patient compliant, microneedles have been proven to be excellent in potentiating robust immune response against a variety of viral and bacterial infections as well as cancer immunotherapy. This robust immune response is attributed to the rich abundance of Langerhans cells, dermal dendritic cells, and other immune machinery in the skin. Microneedles utilize biodegradable polymers and innovative modifications to the polymers to achieve microneedles with tunable properties.

Additionally, microneedles have the potential to be manufactured on a large scale using both the conventional casting as well as non-casting methods. Innovation in the fabrication of microneedles using 3-D printing has paved the way for the potential of personalized vaccination and its large-scale production. Microneedles manufactured using sustained-release polymers or embedded with microparticles or nanoparticles may also eliminate the need to get multiple vaccine shots. Even though there are a few roadblocks, microneedle vaccines have a very bright future and will significantly change how the world gets vaccinated. With the continuous research efforts to overcome the roadblocks, microneedles-based immunizations could play a central role in the way vaccines are administered in the future.

## Figures and Tables

**Table 1:** Diseases, Marketed Vaccines, and their routes.

Route	Vaccine	Disease
Oral	Dukoral^®^, Shanchol™, and Euvichol^®^	Cholera
	Rotarix^®^, RotaTeq^®^	Rotavirus
	Typhim Vi^®^	Typhoid
	Adenovirus type 4 and type 7 vaccine	Adenovirus
Nasal	FluMist^®^	Influenza
IM	Daptacel^®^, Infanrix^®^	Diphtheria, tetanus, pertussis (DTaP)
	Pfizer-BioNTech *COVID*-19 *Vaccine*, Moderna *COVID*-19 Vaccine, Covishield	COVID-19
	Havrix^®^ (Hepatitis A), Engerix^®^ (Hepatitis B); Twinrix^®^	Hepatitis A, Hepatitis B
	Gardasil^®^ 9	Human papillomavirus (HPV)
	Menactra^®^, Trumenba^®^, Bexsero^®^	Meningococcal
SC	M-M-R^®^ II	Measles, mumps, and rubella (MMR)
	Varivax^®^	Varicella (Var)
Intradermal	BCG Vaccine	Tuberculosis

**Table 2:** Companies developing microneedles for vaccine delivery.

Company	Type of Microneedle	Disease	Company Website
Micron Biomedical	Dissolving microneedle	Inactivated rotavirus	[79]
3M (Kindeva)	Hollow microneedle	Cancer vaccines	[80]
BD Technologies (BS Soluvia)	Stainless steel microneedles	Influenza	[81]
Flugen	Metal microneedles	Influenza	[82]
Debiotech	Hollow microneedles	COVID-19	[83]
Verndari (Vaxipatch)	Stainless steel microneedle	Influenza, COVID-19	[84,85]
Nanopass (MicroJet^TM^)	Silicon microneedles	Influenza, Polio, Varicella-Zoster, Cancers, Hepatitis B, COVID-19	[86]
BioSerenTach Inc.	Dissolving microneedles	Vaccine	[87]
Sorrento therapeutics (Sofusa^®^)	Nanotopographical imprinted microneedles (coated)	Immuno-oncology	[88]
Vaxxas (Nanopatch^TM^)	Coated microneedles array patch	Influenza, COVID-19	[89]
Quadmedicine	Dissolving microneedles	Influenza, Canine Influenza	[90]
Vaxess	Dissolving microneedles	Influenza, COVID-19, skin cancer	[91]
Raphas	Dissolving microneedles	HPV, Polio, Tdap, HBV, IPV, and Hepatitis B	[92]

## Appendix A

### Appendix A.1. PRISMA Forms for This Narrative Review

**Table A1:** Literature Review.

Protocol and registration: NA
Eligibility criteria: All published studies with mesh terms: microneedle, skin patch, and vaccine delivery
Information sources: NCBI and Web of Science
Search: https://pubmed.ncbi.nlm.nih.gov/?term=Microneedle+and+vaccine+delivery&sort=date&size=200 https://mjl.clarivate.com/search-results; Accessed date: 13 November 2020, updated on 18 February 2021
Study selection: Narrative review of current literature.
Data collection process: PubMed and Web of Science. First search yielded around 400 papers that were reviewed carefully for inclusion in this narrative review
Data items: 400 papers went through selection for suitability and inclusion in this narrative synthesis
Data collection process: PubMed and Web of Science. First search yielded around 400 papers that were reviewed carefully for inclusion in this narrative review. Additional search included websites of companies with microneedles R & D
Risk of bias in individual studies: NA. Only two clinical trials we available at the time of this study NCT02438423 and NCT03207763. Summary measures: Published paper describing the principles of vaccine delivery routes were included with special focus on studies describing microneedles fabrication, utility and challenges are included
Synthesis of results: The utility of microneedles for vaccine delivery was the basis for this narrative review. This thematic review discusses the current microneedles skin patches state of the art and the potential translation for vaccine delivery

### Appendix A.2. PRISMA for Clinical Trials Using Microneedles

**Table A2:** First study: Inactivated Influenza Vaccine Delivered by Microneedle Patch or by Hypodermic Needle. https://clinicaltrials.gov/ct2/show/NCT02438423. Accessed on 13 November 2020, updated on 18 February 2021.

Protocol and registration: NCT02438423
Eligibility criteria: 18 Years to 49 Years healthy adults
Information sources: Clinical Trials.gov
Search: https://clinicaltrials.gov/ct2/show/study/NCT02438423; Accessed date: 13 November 2020
Study selection: Interventional, Randomized, Phase I Placebo controlled Study of The Safety, Reactogenicity, Acceptability and Immunogenicity of Inactivated Influenza Vaccine Delivered either by Microneedle Patch or by Hypodermic Needle.
Data collection process: This is a single center, partially blinded, randomized phase I study in which healthy adult subjects (ages 18–49) will receive either inactivated influenza vaccine (IIV) (either by microneedle patch or hypodermic needle) or placebo (by microneedle patch) (https://clinicaltrials.gov/ct2/show/study/NCT02438423; Accessed date: 13 November 2020)
Data items: 100 participants recruited and allocated to the following interventional groups: 25 participants in Inactivated Influenza Vaccine (IIV) Delivered by Microneedle (MN) Patch by Study Staff 25 participants in IIV Delivered IM by Study Staff 25 participants in IIV Delivered by MN Patch by Subject 25 participants in Placebo MN Patch by Study Staff
Risk of bias in individual studies: NA, single blinded study includes equal number of males and females
Summary measures: Primary out comes: Occurrence of Solicited Injection Site and Systemic Reactogenicity on the Day of Study Product Administration Through 7 Days After Administration. And Occurrence of Study Product-related Serious Adverse Events From D0 Until D180 (+/− 14 Days) After Study Product Administration Secondary outcome: Geometric Mean Titer (GMT) of HAI Antibody Approximately 28 Days Following Receipt of IIV Delivered by Microneedle Patch or by Hypodermic Needle (Both Vaccines Administered by Study Staff). And Percentage of Subjects Achieving Seroprotection (Defined as a HAI Antibody Titer of 1:40 or Greater) Approximately 28 Days Following Receipt of IIV Delivered by Microneedle Patch or by Hypodermic Needle (Both Vaccines Administered by Study Staff).
Synthesis of results: Published paper “The safety, immunogenicity, and acceptability of inactivated influenza vaccine delivered by microneedle patch (TIV-MNP 2015): a randomized, partly blinded, placebo-controlled, phase 1 trial” The Lancet, 2017, DOI: 10.1016/S0140-6736(17)30575-5. Conclusion: use of dissolvable microneedle patches for influenza vaccination was well tolerated and generated robust antibody responses.

**Table A3:** Second study: Microneedle Patch Study in Healthy Infants/Young Children. (https://clinicaltrials.gov/ct2/show/NCT03207763; Accessed date: 13 November 2020).

Protocol and registration: NCT03207763
Eligibility criteria: 6 Weeks to 24 Months (Child)
Information sources: Clinical Trials.gov
Search: https://clinicaltrials.gov/ct2/show/study/NCT03207763; 13 November 2020, updated on 18 February 2021
Study selection: Interventional, Non-Randomized, A Study to Evaluate the Safety, Reactogenicity, and Acceptability of a Placebo Microneedle Patch in Healthy Infants and Young Children.
Data collection process: Microneedles can be prepared as a low-cost patch that is simple for patients to apply for vaccine delivery targeting the many antigen-presenting cells present in the skin. Data regarding the safety, reactogenicity, tolerability, and acceptability of a microneedle patch in children are lacking. The goal of this study is to evaluate the safety, reactogenicity, and acceptability of placement of a placebo microneedle patch to the skin of children.
Data items: 33 participants recruited and allocated to the following interventional groups: Cohort 1: Eight Participating infants and children receiving Microneedle Formulation 1. Children had a microneedle patch initially applied to the skin overlying the shoulder blade. If the first patch was well tolerated without halting criteria having been met, participants could opt to have two additional microneedle patches applied to the upper arm, forearm, wrist and/or thigh. Cohort 2: 25 Participating infants and children receiving Microneedle Formulation 2. Children had a microneedle patch initially applied to the skin overlying the shoulder blade. If the first patch was well tolerated without halting criteria having been met, participants could opt to have two additional microneedle patches applied to the upper arm, forearm, wrist and/or thigh.
Risk of bias in individual studies: NA
Summary measures: Primary out comes: Number of Participants With Placebo Microneedle Patch-related Serious Adverse Events (SAE). Number of Participants With Grade 3 Placebo Microneedle Patch-related Solicited Adverse Events (AE). Number of Participants With Solicited Application Site Reactogenicity Events. Secondary outcome: Number of Participants With Grade 3 Placebo Microneedle Patch-related Unsolicited Adverse Events. Number of New-onset Medical Conditions (NOMC). Acceptability of Vaccination Methods. Time frame: Day 1, Day 2, Day 8, Final Visit (Day 27–38)
Synthesis of results: No adverse events were recorded for any of the 33 participating infants. Study is not published
